# Physically active academic lessons and effect on physical activity and aerobic fitness. The Active School study: A cluster randomized controlled trial

**DOI:** 10.1016/j.pmedr.2018.12.009

**Published:** 2018-12-28

**Authors:** Per Helge Seljebotn, Ingrid Skage, Anette Riskedal, Marta Olsen, Silje Eikanger Kvalø, Sindre M. Dyrstad

**Affiliations:** a Department of Physical Therapy and Occupational Therapy, Municipality of Stavanger, 4016 Stavanger, Norway; b Department of Education and Sport Science, University of Stavanger, 4036 Stavanger, Norway; c Department of children, youth and education, Municipality of Stavanger, 4036 Stavanger, Norway; d Department of Public Health, University of Stavanger, 4036 Stavanger, Norway

**Keywords:** Child health, Physical fitness, Teaching methods, Physical activity

## Abstract

The Active School program was designed to positively impact health and academic-related outcomes in school. The core intervention component was physically active academic lessons, a teaching activity that combines physical activity and educational content. The purpose of this study was to investigate the effect of a 10-month, cluster-randomized controlled trial on physical activity level and aerobic fitness conducted in the city of Stavanger, Norway, in 2014–15. The physical activity level during physically active academic lessons was also studied. A total of 447 children (9–10 years) participated. The weekly intervention consisted of physically active academic lessons, physically active homework and physically active recess. Physical activity level and aerobic fitness were measured objectively by accelerometry and a 10-minute interval running test. Intervention effects were found for time in moderate to vigorous physical activity (MVPA) (adjusted mean difference of 8 min/day, 95% CI: 3.4–13, p < 0.001) and total physical activity (60 counts/min, 95% CI: 15–105, p = 0.009). Children with low aerobic fitness increased their running distance compared to controls (d = 0.46; p = 0.001). During physically active academic lessons children spent 26% of the time in MVPA, which was comparable to physical education lessons. The Active School program successfully increased physical activity for the intervention group and aerobic fitness for the least fit children. The activity level during physically active academic lessons was as high as in physical education lessons. Clinicaltrail.gov ID identifier: NCT03436355.

## Highlights

• The core intervention component was outdoor physically active academic lessons.
• Physically active academic lessons improved children's physical activity levels.
• The intervention improved aerobic fitness among children with low aerobic fitness.
• These lessons were comparable to physical education, in terms of physical activity.
• A single lesson accumulated 20% of the recommended daily physical activity level.

## 1 Introduction

Evidence suggests that there are positive associations between physical activity (PA), fitness, cognition and academic achievement (Donnelly et al., 2016). Findings supporting the importance of PA for academic learning, and not just health, have increased the interest in PA among politicians and decision makers. Schools are a very promising setting for PA promotion since the majority of potential participants are reached regardless of socioeconomic status, and many school-based interventions on PA and fitness have been found to be effective (Cohen et al., 2015; Dobbins et al., 2013; Kriemler et al., 2011). Unfortunately, there are barriers to the implementation of school-based PA initiatives (Naylor et al., 2015), with lack of time being most consistently cited. Introducing interventions that are complementary to teacher-related activities is therefore of importance in the real-world setting. In physically active academic lessons, hereafter referred to as physically active lessons, teachers combine academic content with PA to increase both learning outcomes and health.

Two reviews (Daly-Smith et al., 2018; Norris et al., 2015) found encouraging evidence of improved PA and educational outcomes following physically active lessons. Mullender-Wijnsma et al. (2016) found that physically active lessons improved mathematics and spelling performance of elementary school children, Riley et al. (2016) stated that integrating movement across the primary mathematics lessons was feasible and efficacious, while Grieco et al. (2016) found that physically active academic games improved time used on academic tasks to a greater extent than sedentary academic games. Physically active lessons are implemented in various school environments and range from spelling relays (Grieco et al., 2016) to exercises that illustrate answers (Martin and Murtagh, 2015) and virtual field trips (Norris et al., 2016). However, most of the recent studies conducted physically active lessons in the classroom (as opposed to outdoors), which can be difficult due to noise and lack of space. One exception is the Active Smarter Kids study which used 3 × 30 min/week physically active lessons, mainly carried out in the schoolyard, as the core intervention. In this study results suggested that low performing children in academic performance benefited the most academically from the intervention (Resaland et al., 2018).

Altogether, relatively few papers report effects from school interventions containing physically active lessons, especially performed outdoors. No studies comparing the PA level during outdoor physically active lessons to other lessons have been found, and studies using objective measures of PA to determine effects from physically active lessons on overall PA levels are wanted (Watson et al., 2017).

The Active School program started in the city of Stavanger, Norway, in 2013 to increase children’s PA level in school to improve health and learning. The core intervention component was physically active lessons. After a successful pilot study in 2013–14 (Skage and Dyrstad, 2016), a 10-month, cluster randomized controlled trial in primary schools was conducted in 2014–15. It was found that increased PA in school tended to benefit children's cognitive functioning (Kvalø et al., 2017). The primary aim of the current study was to report effects of the Active School program on objectively measured PA level and aerobic fitness. In addition, the PA level during the 45-minute physically active lessons was compared to ordinary classroom lessons and physical education (PE) lessons.

## 2 Methods

### 2.1 Participants

**Fig. 1:**
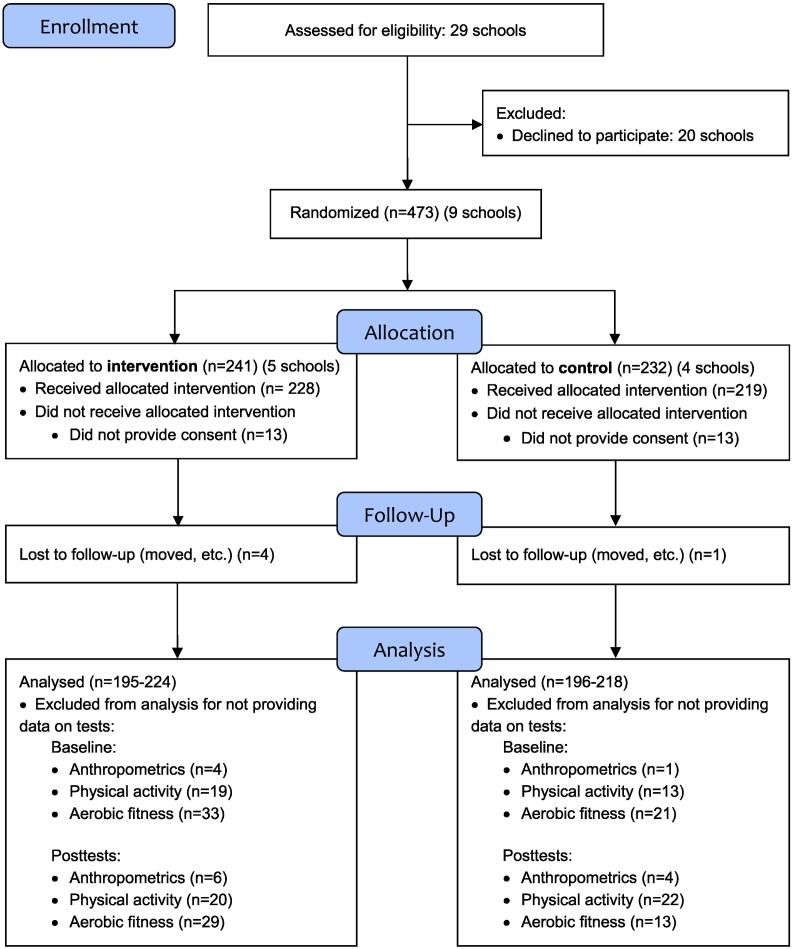
The consort flow diagram of the Active School study.

The Active School study was a 10-month school-based cluster randomized controlled trial conducted in 2014–15. All 29 primary schools in the municipality of Stavanger, Norway, were invited and nine schools agreed to participate. A total of 473 children (5th grade, 9–10 years old) were invited, and 447 (95%, 219 girls and 228 boys) participated in the study (Fig. 1).

The nine schools were matched into two groups stratified by the number of children participating in extracurricular sports, the socioeconomic status in the surrounding school area, size and participation in the “Physical Activity Leader Program” (a separate program that focuses on increasing PA and preventing bullying during recess). The computer program “Researcher Randomizer” (Urbaniak and Plous, 2013) was used to randomize the two groups into intervention and control groups.

### 2.2 Instruments

#### 2.2.1 Anthropometrics

Weight, height and waist circumference were measured by the school nurse according to national standards (Norwegian Institute of Public Health, 2014). Body mass index (BMI) was calculated as weight/height^2^ (kg/m^2^). Age and gender specific BMI cut-off values were used to categorize the children as being underweight, normal weight or overweight/obese (Cole et al., 2000).

#### 2.2.2 Physical activity

Physical activity was measured using accelerometry (ActiGraph GT1M/GT3X/GT3X+, LLC, Pensacola, Florida, USA). The children were asked to wear the accelerometer on the right hip for seven consecutive days, removing it only during water-based activities (e.g., swimming) and while sleeping. Data were considered valid if a child had at least two days with a wear time of ≥480 min/day accumulated between 06:00 and 24:00. There is no consensus of which threshold to use but many previous investigations have used this cut-off (Dencker et al., 2012). Periods of ≥20 min of zero counts were defined as non-wear time. Data were collected in 10-s epochs. These criteria were the same as used in the Physical Activity among Norwegian Children Study (Dalene et al., 2018). Registration started the second day of wearing the monitors to avoid excessive activity likely to occur the first day of wearing the accelerometers. Outcomes for PA levels were 1) sedentary time in min/day (0-100 counts per minute [cpm]), 2) light-intensity PA in min/day (101–2295 cpm), 3) moderate-to-vigorous intensity PA (MVPA) min/day (≥2296 cpm), 4) total PA in cpm, and 5) number of steps per day. The cut-off points were defined by Evenson et al. (2008). Although there is no consensus regarding cut-off points (Dencker and Andersen, 2008), the Evenson cut-points have shown acceptable classification accuracy for all four activity intensities among children (Trost et al., 2011). All accelerometry was analyzed using ActiLife, v. 6.12.0 (ActiGraph Corporation, LLC, Pensacola, FL, USA). The collection of baseline accelerometer data was conducted before the intervention started (August 2014). Post-intervention accelerometer data were collected at the end of the school year (April-June 2015). Schools that gave detailed descriptions of timetables during accelerometer wear time were included in a separate analysis of PA in classroom lessons, PE lessons and physically active lessons. Eighty-seven children (37 boys) were included in this analysis.

#### 2.2.3 Aerobic fitness

Aerobic fitness was assessed by a 10-minute interval running test. The advantages of this test are described in Andersen et al. (2008). The test was organized as follows: A 20 m lane was marked with two parallel lines on the floor of a gym hall. Between 10–22 children ran simultaneously back and forth between these end lines, touching the floor with a finger behind the lines every time they turned around. After 15 s, they rested for 15 s before they ran for another 15 s. This test lasted 10 min, and the running distance was used the outcome measure for aerobic fitness. One adult test assistant per child counted the number of lengths completed. The children completed a 10-minute standardized warm-up before the test. A familiarization trial was performed at least one week before the main test.

### 2.3 Procedure

The intervention was led by teachers in the intervention schools and consisted of one primary component (physically active lessons) and two secondary components (physically active homework and physically active recess). The main component, physically active lessons, was conducted 2–3 times per week, on days without physical education or other curricular PA. All lessons lasted 45 min. The lessons were held mainly outdoors and included games, relays and quizzes with curricular questions from several theoretical subjects. For the other two study intervention components, teachers were encouraged to assign homework in physical education (10 min/day) and offer inspiration for physically active recess (10 min/day).

The intervention was intended to increase the amount of PA by 190 min/week, giving a total of 325 min/week of PA. Control schools were asked to continue their normal routine, which included approximately 135 min/week of PA.

Teachers were asked to organize the physically active lessons using the following guidelines: a) each lesson should include at least 15 min of moderate to vigorous intensity, b) lessons should be easily organized and adapted to avoid unnecessary waiting, c) lessons should include both competitive and noncompetitive elements, and d) lessons should be enjoyable activities that included all the children. To further improve the quality of the physically active lessons, a quality framework were stated at the back of the physically active lesson form, and included tips of how differentiation, autonomy, collaboration, enjoyment and high activity level could be ensured.

To assist and support intervention teachers, one primary and one secondary contact person from the Active School project team was assigned to each intervention school. Contact persons attended meetings and regularly visited participating teachers and classes throughout the school year (1–4 visits per month, depending on requests from the schools). One pre-intervention seminar and one midway seminar were arranged for the teachers to give information about the program and provide support. New physically active lessons were shared between intervention schools through a web site.

### 2.4 Data analysis

Power calculations were based on total PA (cpm), to detect an effect size (Cohen's d) of 0.47. A total of 300 children would provide a power of β = 0.80 to detect relevant changes (repeated measures) between participants in the intervention and control groups.

Preliminary analyses were conducted to ensure no violation of the assumptions of normality and linearity. Mann-Whitney was used to detect differences in MVPA and steps due to a positive Levene's test. A variance component analysis indicated that the variance between schools were low for the primary outcomes: 3.1% in children's moderate to vigorous physical activity, 3.9% in children's total physical activity level (counts/min) and 3.6% in children's running distance (aerobic fitness). Due to low variance between schools in these outcomes, multilevel analysis was not considered necessary. The effectiveness of the intervention was assessed using mixed ANCOVA and ANOVA repeated measures analysis with aerobic fitness and PA as dependent variables, with time (baseline and post-intervention) as the within variable, group (intervention and control group) as the between variable, and in some analyses, gender as the between subject variable. Gender was entered as covariate when appropriate. Cohen's d was calculated to express the distance between two means in standard deviations (effect size). A p-value of less than 0.05 was considered statistically significant. All statistical analyses were performed using SPSS 21 (IBM Corporation, Somers, NY, USA).

## 3 Results

### 3.1 Anthropometric characteristics

Descriptive statistics of children's anthropometric characteristics at baseline are provided in Table 1. There were no differences between intervention schools and control schools in any variables. No significant intervention effect on body mass index (BMI) or waist circumference was found. Data for BMI is shown in Table 2.

**Table 1:** Children's anthropometric characteristics at baseline.

		Control n = 219	Intervention n = 228	p
Girls		111 (51%)	109 (48%)	p = 0.64
Boys		108 (49%)	118 (52%)	
Weight (kg)		35.7 (8.2)	36.5 (7.7)	p = 0.24
Height (cm)		143.0 (6.7)	143.6 (6.3)	p = 0.28
BMI (kg/m^2^)		17.3 (2.9)	17.6 (3.0)	p = 0.37
Waist circumference (cm)		61.9 (7.3)	62.8 (7.6)	p = 0.19
Weight categories^1^	Underweight	32 (14.7%)	28 (12.5%)	p = 0.46
	Normal weight	151 (68.3%)	156 (68.8%)	
	Overweight/obese	37 (17.0%)	42 (18.8%)	

BMI = Body mass index. Mean (percent or standard deviation).

1 Weight categories according to Cole et al. (2000) and Cole et al. (2007).

**Table 2:** Intervention effects on physical activity parameters with change scores.

		Control, n = 188 (♀ = 96, ♂ = 92)			Intervention, n = 189 (♀ = 91, ♂ = 98)			Change	Intervention effect^2^		
		Baseline (SD)	Post-intervention (SD)	Change baseline to post-intervention^1^ [95% CI]	Baseline (SD)	Post-intervention (SD)	Change baseline to post-intervention^1^ [95% CI]	Mean difference between groups [95% CI]	F	p	d
Sedentary (min/day)	All	471⁎ (55)	486 (58)	16 [6, 25]⁎⁎	480 (60)	482 (69)	2 [−7, 11]	−13 [−26, 0]	4.09	0.054	0.21
	Girls	468⁎ (51)	493 (56)	24 [13, 36]⁎⁎	491 (55)	487 (56)	−4 [−16, 8]	−29[−45, −12]	11.53	**0.001**	**0.50**
	Boys	473 (59)	479 (60)	6 [−8, 21]	470 (63)	478 (80)	8 [−6, 22]	3 [−18, 23]	0.27	0.805	0.10
Light activity (min/day)	All	228 (32)	214 (34)	−14 [−19, −9]⁎⁎	225 (38)	216 (37)	−9 [−15, −4]⁎⁎	−5 [−12, 3]	1.43	0.232	0.12
	Girls	233 (31)	217 (33)	−16 [−24, −8]⁎⁎	230 (37)	217 (36)	−13 [−21, −5]⁎⁎	3 [−8, 14]	0.30	0.587	0.08
	Boys	223 (33)	211 (35)	−12 [−19, −5]⁎⁎	220 (38)	214 (39)	−6 [−13, 1]	7 [−4, 17]	1.37	0.244	0.17
MVPA (min/day)	All	68⁎ (26)	68 (23)	0 [−4, 3]	61 (19)	69 (24)	8 [4, 11]⁎⁎	8 [3, 13]	11.02	**0.001**	**0.34**
	Girls	59 (18)	60 (18)	1 [−3, 5]	57 (18)	63 (19)	6 [2, 10]⁎⁎	5 [−1, 11]	2.70	0.102	0.24
	Boys	77 (29)	75 (24)	−2 [−8, 3]	66 (20)	75 (29)	9 [4, 15]⁎⁎	11 [4, 19]	8.67	**0.004**	**0.43**
CPM (axis 1)	All	622⁎ (199)	626 (230)	8 [−24, 40]	584 (176)	659 (271)	69 [37, 101]⁎⁎	60 [15, 105]	7.02	**0.008**	**0.27**
	Girls	580 (147)	578 (189)	−2 [−43, 39]	552 (155)	623 (211)	71 [29, 113]⁎⁎	73 [14, 132]	5.94	**0.016**	**0.40**
	Boys	654 (221)	672 (259)	18 [−31, 67]	622 (190)	688 (265)	67 [19, 114]⁎⁎	48 [−21, 116]	1.95	0.164	0.20
Steps per day	All	11476 (2940)	11160 (2696)	−313 [−737, 110]	10537 (2245)	11175 (2959)	636 [214, 1058]⁎⁎	940 [341, 1540]	9.69	**0.002**	**0.32**
	Girls	10645 (2353)	10447 (2327)	−198 [−776, 381]	10299 (2011)	10649 (2344)	350 [−244, 944]	548 [−282, 1377]	1.70	0.194	0.19
	Boys	12343 (3239)	11904 (2861)	−439 [−1060, 183]	10758 (2430)	11664 (3372)	906 [303, 1508]⁎⁎	1328 [459, 2198]	9.39	**0.003**	**0.45**
BMI^3^	All	17.3 (2.9)	17.5 (2.9)	0.2 [0.1, 0.3]	17.5 (3.0)	17.8 (3.0)	0.3 [0.2, 0.4]	0.1 [−0.4, 0.3]	2.06	0.152	0.14
	Girls	17.5 (3.1)	17.7 (3.1)	0.2 [0.0, 0.3]	17.5 (2.8)	17.9 (2.8)	0.3 [0.2, 0.5]	0.2 [−0.1, 0.4]	2.13	0.146	0.20
	Boys	17.2 (2.8)	17.4 (2.8)	0.2 [0.1, 0.4]	17.5 (3.1)	17.8 (3.1)	0.3 [0.1, 0.4]	0.1 [−0.2, 0.3]	0.30	0.584	0.08

1 Change baseline to post-intervention (all) was adjusted for gender.

2 Significant p-values (p < 0.05) and their effect sizes in bold. d=Cohen's d.

3 n = 431. Control, n = 213 (♀ = 109, ♂ = 104). Intervention, n = 218 (♀ = 105, ♂ = 113).

⁎ Statistically significant differences between control and intervention at baseline, p < 0.05.

⁎⁎ p < 0.01; pairwise comparisons post hoc, using the Bonferroni correction for multiple comparisons.

### 3.2 Physical activity level and sedentary behavior

Small to medium effect sizes were found on sedentary behavior (reduced sedentary time), cpm, MVPA and steps, all in favor of the intervention group (Table 2). The intervention group had a 12% increase in cpm, while the control group showed no change. The control group had no significant change in MVPA, while the intervention group had a 13% increase. No intervention effect on light activity was found.

Secondary analyses showed that the intervention effect on sedentary behavior and cpm was only significant for girls (net effects: −28 min, 73 cpm), while the intervention effect on MVPA and steps was only significant for boys (net effects: 11 minutes MVPA, 1345 steps).

### 3.3 Aerobic fitness

Both the intervention and the control group significantly increased their running distance from baseline to post-intervention (4% in both groups), but no overall significant effect of the intervention was found.

A secondary analysis compared intervention effects in the lowest scoring tertile at baseline, revealing significant intervention effects in favor of the intervention group (Table 3). Boys in the lowest tertile in the intervention group had almost twice the increase in running distance, as compared to the corresponding boys in the control group (p = 0.006). There were no significant differences between any of the other tertiles (Table 3).

**Table 3:** Change in running distance for all and by tertile from baseline to post-test, N = 364.

	Control n = 186 (♀ = 97, ♂ = 89)			Intervention n = 178 (♀ = 81, ♂ = 97)			Intervention effect^2^		
	n	Baseline	Change from baseline (m)	n	Baseline	Change from baseline (m)	F	p	d
First tertile (meters)	64	874 (56)	37	58	893 (50)	65	6.2	**0.01**	**0.46**
Second tertile (meters)	61	971 (39)	44	61	997 (38)	31	1.8	0.19	
Third tertile (meters)	61	1070 (57)	29	59	1093 (61)	25	0.1	0.76	
All	186	970^1^ (96)	36	178	995 (96)	40	0.4	0.52	

Mean (standard deviation).

1 Significant differences between intervention and control at baseline, p < 0.05.

2 Significant p-values (p < 0.05) and their effect sizes in bold. d = effect size (Cohen's d).

### 3.4 Physical activity level during physically active lessons

During the 45-minute physically active lessons, children spent just as much time in MVPA (27% of the lesson) and completed 17% more steps than in the PE lessons. Children were sedentary 78% of the time during academic classroom lessons, which was twice as much as during PE- and physically active lessons (Table 4).

**Table 4:** Physical activity (PA) variables during three different school lessons (each lasting 45 min), n = 87.

	Physically active lessons	Physical education (PE) lessons	Academic lessons
Sedentary activity (min)	16 (6)⁎	18 (5)	34 (5)⁎⁎
Light PA (min)	17 (4)	16 (3)	9 (4)⁎⁎
MVPA (min)	12 (4)	11 (4)	2 (2)⁎⁎
Vigorous PA (min)	6 (2)	6 (3)	0 (1)⁎⁎
Counts per minute	1559 (470)	1481 (508)	277 (193)⁎⁎
Steps (number)	1699 (544)⁎⁎	1451 (451)	387 (244)⁎⁎

Mean (standard deviation).

⁎ Different from the other lessons (p < 0.05).

⁎⁎ Different from the other lessons (p < 0.001).

Comparing MVPA during physically active lessons to overall daily MVPA, and MVPA during school time indicated that each physically active lesson contributed to 16% of overall daily MVPA and 30% of MVPA during school time.

## 4 Discussion

The purpose of this study was to evaluate changes in children's PA level and aerobic fitness after a 10-month school intervention program in which physically active lessons was the core intervention component. An overall intervention effect was increased PA level and reduced sedentary time. No overall effect of the intervention was found on aerobic fitness. However, the least fit tertile in the intervention group had a moderate intervention effect on the running distance compared with the corresponding tertile in the control group. During the physically active lessons, children's PA levels were as high as in PE lessons.

The current study showed that the least aerobically fit children gained the most from the intervention, which is not surprising since these children have the greatest potential for improvement. In our study a 10% increase in total PA level (cpm) was enough to increase fitness for the 1/3 least aerobically fit children. This is an encouraging finding suggesting that the least fit children might benefit from even modest increases in daily PA. The increase in aerobic fitness among the least fit is also consistent with studies finding a moderate to strong (β = 0.63) association between change in daily PA (cpm) and aerobic fitness in children with initially low levels of PA (Kristensen et al., 2010).

The intervention group had a 15% increase in MVPA, while subgroup analysis showed that girls had a greater reduction in sedentary time, while boys had a greater increase in MVPA. The difference between boys and girls with respect to changes in MVPA could explain why boys in the least fit tertile had a greater increase in running distance than girls, and also agrees with results of Bailey et al. (2012), who found that boys were more involved in MVPA than girls.

As shown in Table 2, children in the control group were significantly less sedentary and more physically active at baseline than children in the intervention group. It could therefore be argued that children in the intervention group were not outperforming the control group at posttest, but that they had ‘caught up’, also called regression to the mean. However, the difference in sedentary behavior between groups were small (less than 2%), and even though children in the control group had a lower fitness than children in the intervention group at baseline (Table 3), it was only the least fit children in the intervention group who increased their fitness.

Data from the present study show that 12 of the 45 min of the physically active lesson were carried out as MVPA, which was the same amount of MVPA as in PE lessons. During physically active lessons, sedentary time was replaced with MVPA, and contributed to 20% (12/60 min) of the recommended amount of daily PA. A systematic review stated that all patterns of PA (sporadic, bouts, continuous) provided beneficial health indicators in school-aged children, and that all intensities of PA should be considered in future work aimed at better elucidating the health benefits of PA in children and youth (Poitras et al., 2016).

Several studies have reported increased PA due to physically active lessons, (see references in Norris et al. (2015)), while only one study was found that included fitness as an effect variable. In the “Fit and academically proficient at school” study (Mullender-Wijnsma et al., 2015), no effects on cardiovascular and muscular fitness were found (de Greeff et al., 2016). In that study, children participated in physically active lessons three times a week, 20–30 min each time. Based on heart rate monitors worn in a subsample in one lesson, the study’s conclusion was similar to our finding: the duration of MVPA in the physically active lessons was comparable to the average time spent in MVPA during a PE lesson. This shows that physically active lessons are a feasible approach to reducing sedentary time and increasing MVPA among schoolchildren.

Considering the intervention's intended 190-minute increase in PA, the effects on MVPA and light activity might seem small. Adding the net effects of light activity (+5 min) and MVPA (+8 min), this daily 14-minute net increase add up to a 70-minute increase per week. The teachers' reports indicated that 73% of the physically active lessons were carried out as planned, data reported in Dyrstad et al. (2018), and around 1/3 of the physically active lessons was needed for instructions and discussions. Even so, the 70 min/week of increased PA, was enough to increase the aerobic fitness for 1/3 of the children with the lowest aerobic fitness. In addition to physically active lessons, the intervention included physically active homework and physically active recess. During the implementation evaluation, it was found that teacher feedback regarding the homework and recess components of the intervention was mixed. Three of the five intervention schools had already incorporated physically active recess. In addition, teachers at one of the schools questioned the need for physically active homework, pointing out that many children were already involved in organized PA during their leisure time. On the other hand, teachers at two of the intervention schools received positive feedback from both children and parents regarding physically active homework, and wanted to continue with it after the intervention period. The main findings from the present project’s process evaluation results were that physically active lessons were considered an appropriate pedagogical method for creating positive variation, and were highly appreciated among both teachers and children (Dyrstad et al., 2018). These findings, combined with the observed effects on PA and fitness, further supports physically active lessons as a feasible intervention in schools.

### 4.1 Limitations

The study has some limitations. First, the PA measurements are only snapshots of the activity level during the specific weeks, and were susceptible to special activities taking place during these weeks. Second, due to the low number of participating schools (N = 9), we did not have the statistical power to use multilevel analysis. However, the variance between schools was low. Third, even though the fidelity was acceptable (75% of the extra PA was reported delivered as planned), implementation of the intervention components varied between schools. Due to the design, teachers should develop, adapt and adjust the intervention components to their local settings. It is therefore not possible to state which components were most effective. However, in a real-world setting, interventions would need to be customized to fit different school settings. It should also be noted that the statistical analyses did not follow the intention-to-treat principle, since children dropping out from the post intervention tests were excluded from analysis in Table 2, Table 3.

## 5 Conclusions

This study showed that a relatively modest intervention implemented in a real-world setting could reduce sedentary behavior and increase PA among children. Furthermore, it was found that a 10% increase in total PA level (measured in counts per minute) was enough to give a moderate effect on running distance for the least aerobically fit children. The PA level during physically active lessons was just as high as during PE lessons. Even though it is not possible to determine the size of the contribution from each of the three different intervention components (physically active lessons, physically active homework and physically active recess), physically active lessons stand out as an effective intervention for reducing sedentary time and increasing PA and fitness in school without reducing academic content. Physically active lessons were a significant contributor to children meeting the PA guidelines, and a single lesson contributed to 20% of the daily recommended amount of PA.

Children with the lowest aerobic fitness are often those who are most difficult to involve in PA during leisure time, and who normally show the greatest gains in health indicators when being physically active. Our findings suggest that integration of physically active lessons in the school curriculum can be a successful way to increase the amount of PA for all children, and that the Active School program can increase aerobic fitness for the children with the lowest aerobic fitness. Physically active lessons are a cost-effective approach to both learning and health since this teaching activity does not take time from other subjects or extend the school day, and it can be implemented by all teachers.

## Human subjects approval statement

Parental written informed consent was collected for all children who also gave their verbal consent before they were included in the study. The project was reported to and approved by the Norwegian Centre for Research Data.

## Competing interests

The authors declare that they have no competing interest.
